# A prospective cohort study of the feasibility and acceptability of depot medroxyprogesterone acetate administered subcutaneously through self-injection^☆^^☆☆^^★^

**DOI:** 10.1016/j.contraception.2016.10.007

**Published:** 2017-03

**Authors:** Jane Cover, Allen Namagembe, Justine Tumusiime, Jeanette Lim, Jennifer Kidwell Drake, Anthony K. Mbonye

**Affiliations:** a PATH, PO Box 900922, Seattle, WA 98109, USA; b PATH, PO Box 7404, Kampala, Uganda; c Uganda Ministry of Health, PO Box 7272, Kampala, Uganda

**Keywords:** Home and self-injection, Self-administration, DMPA-SC, Sayana® Press, Injectable contraception

## Abstract

**Objectives:**

Evidence on contraceptive self-injection from the United States and similar settings is promising, and the practice may increase access. There are no published studies on the feasibility of contraceptive self-injection in sub-Saharan Africa to date. The purpose of this study was to assess feasibility of subcutaneous depot medroxyprogesterone acetate self-injection in Uganda, with specific objectives to (a) measure the proportion of participants who self-injected competently, (b) measure the proportion who self-injected on time 3 months after training (defined conservatively as within 7 days of their reinjection date) and (c) assess acceptability.

**Study design:**

In this prospective cohort study, 380 18–45-year-old participants completed self-injection training by licensed study nurses, guided by a client instruction booklet, and practiced injection on prosthetics until achieving competence. Nurses supervised participants' self-injection and evaluated injection technique using an observation checklist. Those judged competent were given a Sayana® Press unit, instruction booklet and reinjection calendar for self-injection at home 3 months later. Participants completed an interview before and after self-injection. Nurses visited participants at home following reinjection dates; during the follow-up visit, participants demonstrated self-injection on a prosthetic, injection technique was reevaluated, and a postreinjection interview was completed.

**Results:**

Of 368 participants followed up 3 months posttraining, 88% [95% confidence interval (CI) = 84–91] demonstrated injection competence, and 95% (95% CI=92–97) reinjected on time, while 87% (95% CI=84–90) were both on time and competent. Nearly all (98%) expressed a desire to continue.

**Conclusions:**

Self-injection is feasible and highly acceptable among most study participants in Uganda.

**Implications:**

The first research results on contraceptive self-injection in sub-Saharan Africa indicate initial feasibility and acceptability of the practice 3 months after women received one-on-one training and a highly visual training and memory aid. Results can inform self-injection programs which aim to increase women's autonomy and access to injectable contraception.

## 1 Introduction

Injectable contraceptives are the most popular modern method in Uganda, representing about 56% of contraceptive use for married women [1]. However, about 25% of all women in Uganda still have an unmet need for modern contraception [1]. Expanding service-delivery options through self-injection may improve injectable access particularly in remote areas — eliminating the need to return quarterly for reinjection by a trained clinic or community health worker — and may offer enhanced confidentiality to women whose partners or social network oppose contraceptive use (although it could also be challenging for these women to store devices and inject at home).

Depo-subQ Provera 104 is a variation of intramuscular depot medroxyprogesterone acetate (DMPA-IM) that contains 104 rather than 150 mg of DMPA administered as a subcutaneous injection (DMPA-SC). DMPA is effective and safe in either form. A recent review of 14 studies concluded that DMPA-SC and DMPA-IM are therapeutically equivalent, with similar side effects profiles [2]. Aggregated data from seven clinical trials of DMPA-IM and two trials of DMPA-SC revealed a first-year failure rate of 2 per 1000 women [3].

Sayana® Press^i^ (DMPA-SC in the Uniject™ injection system^ii^) received regulatory approval in Europe in 2012 and Uganda in 2014. The Uniject™ is a small, prefilled, autodisable device that requires minimal training for use. Acceptability studies of Sayana® Press found that most injectable clients and providers in Uganda and Senegal preferred Sayana® Press over DMPA-IM [4], [5], [6]. In 2015, the UK Medicines and Healthcare products Regulatory Agency approved relabeling Sayana® Press to support self-injection [7]. As of mid-2016, a similar update is under review by the Uganda National Drug Authority and in a few other countries as well.

Although self-injection of contraceptives is relatively new, a limited number of studies have been conducted. Pfizer Inc. tested the safety and efficacy of self-injection of DMPA-SC in a prefilled syringe involving 6279 women-cycles of exposure via self-injection [8]. Women reported self-injection was convenient and easy. There were no pregnancies among women practicing self-injection in these original clinical trials or studies in Florida [9], Scotland [10] and New York [11], although it is important to note that these studies involved limited women-months of exposure due to small samples and short time frames. Self-injection of another injectable contraceptive delivered in a prefilled Uniject™ device, Cyclofem®, was also tested at health clinics in Brazil nearly 20 years ago; 90% of women who self-injected did so correctly, although nearly half of the women invited to do so opted not to self-inject [12]. To date, there have been no published studies on the feasibility of contraceptive self-injection in sub-Saharan Africa.

The purpose of our study was to assess feasibility and acceptability of self-injection with DMPA-SC in Uniject™ in Uganda. The two primary objectives were to measure the proportion of study participants who demonstrate competent self-injection technique 3 months after training and to measure the proportion who self-inject on time. Our secondary objective was to assess the acceptability of self-injection reflected in the proportions who expressed a desire to continue with self-injection and who reported that they were likely or very likely to recommend the practice to others.

## 2 Material and methods

### 2.1 Study design and procedures

This prospective cohort study was conducted in three public facilities in Mubende District and one NGO clinic and two public facilities in Gulu District of Uganda from June to December 2015. These sites included two rural facilities, two urban hospitals, an urban facility that serves youth and does outreach to rural areas and a periurban facility located near a college. Female participants were 18 to 45 years of age and eligible to receive injectable contraception per standard of care guidelines. All had sought care at the clinic and decided to use injectable contraception prior to recruitment, and all provided signed informed consent. Anyone who was pregnant, did not reside permanently in the area, felt unwell on the day of enrollment or did not speak the primary language of the area was excluded from the study. The study received institutional review board (IRB) approval from the PATH Research Ethics Committee and the Mulago Hospital IRB in Uganda.

Study procedures were implemented by licensed nurses who had been trained in Sayana® Press administration, research ethics (including Good Clinical Practice), interviewing, and how to train and counsel women for self-injection.

Study participants were trained one-on-one by study nurses guided by a client instruction booklet (Appendix A) [13] as a visual aid and practiced the injection on a prosthetic device until competence was achieved (study definition of competence is provided in Section 2.2). Client instruction booklets were designed for low-literacy audiences and pretested in Uganda to maximize usability. Training included calculation of injection dates, review of DMPA side effects and protection against HIV, safe home storage and appropriate disposal practices (e.g., placing the used device in an impermeable container before disposing of it in the latrine, retaining the container).

At enrollment and after training, women self-injected under the supervision of a nurse. Their injection technique was evaluated using an observation checklist (Appendix B) [14]. Participants completed two structured interviews on the day of enrollment: one baseline interview before and one postinjection interview. Those judged to be competent, consistent with the checklist, were given one Sayana® Press unit, the client instruction booklet and a reinjection calendar with the reinjection date marked. Three months after the observed self-injection, participants were instructed to self-inject independently at home.

At follow-up, each self-injecting participant was visited at home by the study nurse at least 7 days following her scheduled reinjection date so that it was feasible to assess whether she had completed her injection on time. The participant demonstrated the injection steps on a prosthetic device without guidance from the study nurse, and her technique was evaluated according to the observation checklist. If she had forgotten to do the injection, the nurse observed and evaluated as she gave herself the injection. If she did not want to self-inject but wished to continue with the method, the nurse administered the injection. Every woman completed a follow-up interview, regardless of whether she self-injected.

### 2.2 Data collection and analysis

Data were collected via structured questionnaires in private face-to-face interviews with each study participant. Interviews were conducted in the local language (or English, if preferred by participants) by female interviewers, and data were entered electronically on Android tablets. The same study nurses who enrolled women conducted the follow-up visits.

A sample size of 380 self-injectors was calculated to achieve a desired precision of 5 percentage points around the point estimate of 80% of women competent in self-injection at 3 months [95% confidence interval (CI)=75%–85%] and a precision of 5 percentage points around the point estimate of 80% of women reinjecting consistent with the schedule. The sample size assumed 10% lost to follow-up and 20% discontinuation of the injectable.

The primary objective of evaluating competency in self-injection had two end points: the percent of women who demonstrated correct self-injection technique consistent with the observation checklist and the percent who reinjected on schedule, i.e., within 1 week of their reinjection date. To qualify as competent, a participant had to successfully demonstrate 5 critical injection steps from the 19-step observation checklist (Appendix B) [14]. The five critical steps were so designated since the omission of any of these steps could lead to an ineffective injection. The five steps are as follows: (a) select an appropriate injection site, (b) mix the solution by shaking vigorously, (c) push the needle shield and port together to activate the device, (d) pinch the skin to form a “tent” and (e) squeeze the reservoir slowly to inject the contraceptive. Women who did not demonstrate competence at the first injection were not given Sayana® Press for independent self-injection at home and were exited from the study. Women who discontinued the injectable (e.g., switched methods or stopped family planning in order to become pregnant) and women lost to follow-up were not included in the denominator for the calculation of the primary outcomes.

Data analysis was performed using STATA version 14.1. 95% CIs for the primary end points were calculated using exact CIs for binomial proportions.

## 3 Results

### 3.1 Participant characteristics

A total of 384 participants enrolled in the study. Two women enrolled were later determined ineligible due to pregnancy (both screening failures at the time of recruitment). Data were lost for one enrolled participant, and one participant did not complete enrollment study procedures. For baseline data analysis purposes, participants consisted of 380 healthy female adults who agreed to use injectable contraception and were willing to try self-injection. Table 1 summarizes the sample characteristics.

### 3.2 Injection competence and adherence to reinjection schedule

Among the 380 self-injectors, 98% demonstrated competence immediately after training. Seven women were lost to follow-up, and five women discontinued the injectable for reasons unrelated to self-injection — leaving 368 participants for follow-up analysis. Among these participants, 88% demonstrated competence immediately and 3 months later (Table 2).iii Among the 360 women who reinjected at 3 months, 95% reinjected themselves on schedule; four women injected between 2 and 4 weeks early, and 14 women injected 2 to 3 weeks late (Table 2). A total of 87% of women who reinjected were both competent and on time. Note that five women demonstrated the injection on themselves rather than a prosthetic since they had not yet self-injected at the time of the follow-up visit.

**Fig. 1:**
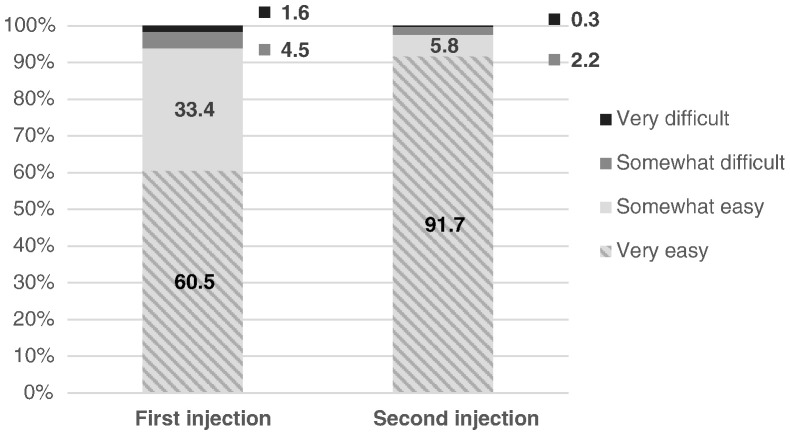
Reported ease of self-injection.

**Fig. 2:**
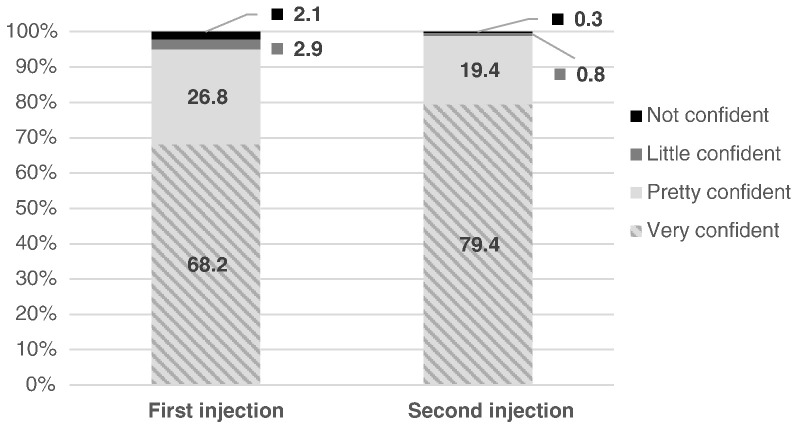
Confidence in ability to self-inject.

With respect to subjective competency, 92% reported that their second injection was “very easy” to do (Fig. 1), an increase from 61% for the first injection. Four out of five (79%) were “very confident” that they gave the second injection correctly, an increase from 68% (Fig. 2). The vast majority of women (96%) reported using the client instruction booklet as a guide for their independent self-injection (data not shown).

### 3.3 Acceptability of self-injection

**Fig. 3:**
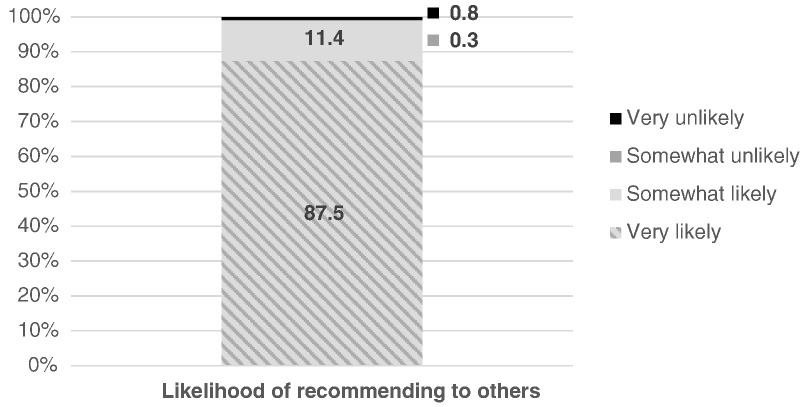
Likelihood of recommending self-injection.

Nearly all of those who gave two injections (98%) expressed a desire to continue with self-injection (data not shown), and 88% reported they were “very likely” to recommend the practice to others (Fig. 3).

### 3.4 Safety

There were no serious adverse events. The most common adverse events were known side effects of DMPA: amenorrhea and other changes in menstrual bleeding patterns, including spotting. Twenty-two women experienced an injection-site reaction (ISR) in the form of a dimple, bruise, blister or nodule, and three women sought treatment or advice for an ISR. All adverse events were mild to moderate in severity.

### 3.5 Storage and disposal

Nearly all women (98%) felt that they were able to keep the device secure (safe from discovery by children or others) until it was time for reinjection, with most (61%) storing it in a handbag (Table 3). Discarding the device in a pit latrine was the preferred disposal strategy (94%), although some returned the device to the clinic or kept it for the study nurse, and two women discarded it with the household garbage. About 7 in 10 women (71%) stored the spent device in an impermeable household container (as instructed during training) until it could be safely discarded.

## 4 Discussion

This study provides the first evidence from sub-Saharan Africa that self-injection of DMPA-SC in Uniject™ is feasible for the vast majority of women after a single one-on-one training session — including women with limited education and younger women. These results echo previous studies on self-injection from developed countries but further demonstrate that self-injection is feasible and desirable in settings where women have relatively less education and access to health information and services.

The results of this study also point to important program and policy implications for potential introduction of injectable contraceptive self-injection [15], [16], which could help increase women's contraceptive access and autonomy. The Sayana® Press manufacturer has announced the intention to register self-injection in an increasing number of countries over the next 1 or 2 years [7], and additional studies of feasibility and impact of the practice are planned or ongoing in Ghana, Democratic Republic of Congo, Nigeria, Uganda, Senegal and Malawi. If and when national drug authorities officially approve self-injection, ministries of health and family planning implementing organizations will need to make evidence-informed decisions about integration of self-injection into routine family planning service delivery.

Our results provide additional context following the recent recommendation of the World Health Organization that self-injection be offered “in contexts where mechanisms to provide the woman with appropriate information and training exist, referral linkages to a healthcare provider are strong, and where monitoring and follow-up can be ensured” [17]. Self-injection competence in this study was achieved for most women in the context of one-on-one training that included review of a booklet geared toward low-literacy audiences and as many practice injections as needed. The time required for training and support of clients in this research study was considerable, and given the many duties of family planning providers, this may be difficult to replicate in daily practice. The provision of supplies to support the training, including saline-filled Uniject™ devices for practice injections, models for injection practice (salt-filled condoms), instruction booklets and calendars, complicates and increases the costs of offering self-injection. A next step for the field will be to design simpler, low-cost but still evidence-based approaches to self-injection practice to rigorously monitor/evaluate actual practice, including the costs, and share those experiences. At the same time, it is important to recognize that time and other costs invested in training clients up front may be offset over the long run by reduced clinic visits for women who continue injecting at home.

Disposal of used devices is another unresolved challenge. Latrine disposal removes the spent device from risk of contact quickly but may not be an ideal strategy given limited global access to sanitation facilities [18] and questionable appropriateness of disposal of medical devices in latrines. Programs should strategize ways to recapture spent devices for incineration without defeating the purpose of self-injection by requiring women to return the device to the clinic. Our results suggest that extra focus during training is needed to encourage more women to secure the device in a locally available, impermeable container prior to elimination [19].

### 4.1 Study limitations

By design, self-injection was not directly observed at the 3-month reinjection time, and it is possible that injections demonstrated on the model may not mirror women's actual practices. Moreover, the five women who self-injected at follow-up rather than injecting on a prosthetic reflect a difference in instrumentation of unknown significance. The small number of cases that differ in this regard is unlikely to influence the results. Because of the timing of the follow-up visit (within the 4-week reinjection window for DMPA) [20], we could not assess whether women would have remembered to self-inject within the window if not for the study nurse follow-up visit. The rationale for conducting follow-up within the window was to ensure that women who had not yet done the injection could receive the injection from the study nurse if desired. The study involved only one unsupervised self-injection; women's recall of injection technique or timing might decrease or increase over time, and their need for support from providers may or may not increase with time/more injections.

An additional limitation is that we did not vary our approach to training clients to self-inject, so it is not possible to assess which specific components of our approach were critical to competent and on-time client reinjection or acceptability — in other words, whether one-on-one training, practice injections or client instruction booklets were critical to our outcomes.

Participants were recruited at public-sector family planning clinics from among clients who had selected injectable contraception. Had there been awareness raising regarding the opportunity to try self-injection, a different family planning clientele and possibly more new users may have been attracted. Feasibility and/or acceptability might vary for clients who typically receive contraceptives from community health workers.

## Figures and Tables

**DMPA-SC (Sayana Press) is delivered in the Uniject injection system.**
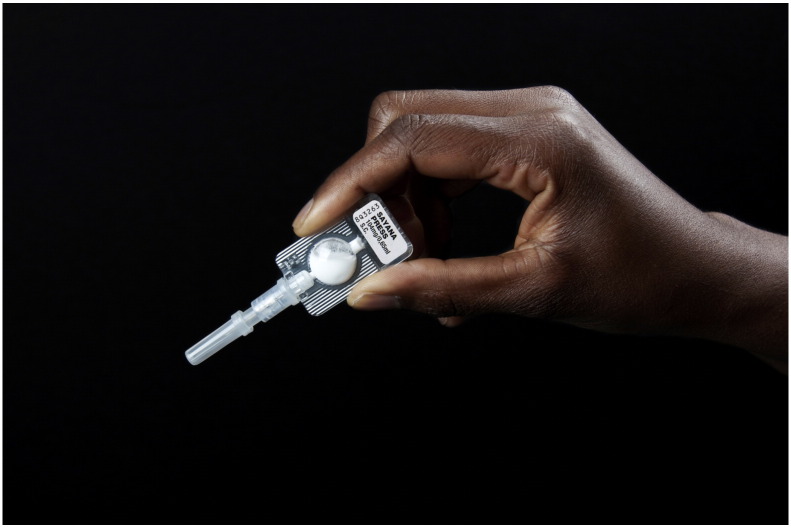


**Table 1:** Baseline characteristics of participants

	Percent or mean	*n*/*N*
Mean age in years	26.0 (SD=6.0)	380
Education		380
None	8.7	33
Primary	56.8	216
Secondary	29.7	113
Postsecondary	4.7	18
Marital status		380
Married and cohabiting	67.6	257
Married, living apart	13.7	52
Single	18.7	71
Mean parity	2.9 (SD=1.9)	380
Contraceptive experience		380
New user of family planning	10.5	40
New user of the injectable	10.3	39
Experienced injectable user	79.2	301
Partner supports family planning use	71.2	269/378
Concerned about privacy at the clinic		379
Not at all concerned	83.9	318
A little concerned	12.9	49
Very concerned	3.2	12
Level of anxiety about self-injection	0.32 (SD=0.51)	377
Number of practice attempts prior to self-injection	2.7 (SD=0.91)	380
Travel time to reach clinic		380
<30 min	54.0	205
30 min–1 h	27.6	105
1–2 h	12.1	46
>2 h	5.1	24
Paid for transport to reach clinic	44.6	169/379
Mean travel expense (in $) if >0	$0.87 (SD = $0.72)	169
Missed work for clinic visit	34.9	132/378

**Table 2:** Injection competence and adherence to reinjection schedule

	%	95% CI	*n*/*N*
Women demonstrating competence immediately posttraining	97.9	95.9–99.1	372/380
Women demonstrating competence 3 months posttraining	88.0	84.3–91.2	324/368
Women who reinjected on schedule (±1 week)	95.0	92.2–97.0	342/360
Reinjection timing:			360
4 weeks early (22–30 days early)	0.6		2
3 weeks early (15–21 days early)	0.3		1
2 weeks early (7–14 days early)	0.3		1
On time (±1 week)	95.0		342
2 weeks late (7–14 days late)	3.3		12
3 weeks late (15–21 days late)	0.6		2
Percent of women reinjecting on schedule and demonstrating competence at follow-up	86.9	83.0–90.2	313/360

**Table 3:** Storage and disposal

	%	*n*/*N*
Device kept secure until use	97.5	355/366
Storage location		364
Handbag	61.3	223
Suitcase	21.2	77
Other	17.6	57
Spent device disposal		354
Returned to clinic	3.39	12
Kept for study nurse	2.26	8
Put in household garbage	0.56	2
Dumped in pit latrine	93.79	332
Stored in container until disposal	71.5	253/354

## Appendix A Supplementary data

Sayana Press self-injection client instruction booklets were used to train and support study participants. Image 1

This observation checklist for Sayana Press injection practice was used to evaluate competence. Image 2

## Footnotes

i Sayana® Press is a registered trademark of Pfizer Inc.

ii Uniject™ is a trademark of BD.

iii The eight women who did not qualify as competent for the first injection did not continue with the study but are included in the denominator for the calculation of percent competent (the primary outcome). Note that since they were not permitted to continue with independent self-injection, these eight cases are not included in the calculation for the percent reinjecting on schedule.
