# Supplementary material for: A prospective cohort study of the feasibility and acceptability of depot medroxyprogesterone acetate administered subcutaneously through self-injection
Source: Contraception. 2017 Mar;95(3):306–11. doi: 10.1016/j.contraception.2016.10.007 (PMC5356471; doi:10.1016/j.contraception.2016.10.007)
Supplement: Supplementary file 2 — This observation checklist for Sayana Press injection practice was used to evaluate competence. [file mmc2.docx]

## Observation checklist for Sayana Press injection practice

Woman number |___|___|___|___|___| Health center number |___|___|

Interviewer code |___|___|___| Date: DD/MM/YY |____|____|____|

**This checklist is used to evaluate Sayana^®^ Press injection practice, using objective performance standards. Give the participant the Self-injection Booklet to use as a guide while she is practicing and giving herself the injection. The first five observations are for practice on a salt filled condom. The sixth observation is for supervised self-injection (SI). For each practice attempt, write S (satisfactory), U (unsatisfactory) or ND (not done) in the box corresponding to each step.**

| **Steps** | Observations | | | | | |
| --- | --- | --- | --- | --- | --- | --- |
|  | 1 | 2 | 3 | 4 | 5 | SI |
| 1. Washes hands |  |  |  |  |  |  |
| 1. **Selects an appropriate injection site and cleans if needed.** |  |  |  |  |  |  |
| 1. Opens the Sayana Press pouch by tearing the notch. |  |  |  |  |  |  |
| 1. Holds the device by the port while mixing. |  |  |  |  |  |  |
| 1. **Mixes the liquid by shaking the device vigorously (about 30 seconds).** |  |  |  |  |  |  |
| 1. Checks to make sure the liquid is mixed and there is no damage to the device. |  |  |  |  |  |  |
| 1. Holds the device with the needle pointing upward during activation. |  |  |  |  |  |  |
| 1. Holds the device by the port while activating. |  |  |  |  |  |  |
| 1. **Pushes the needle cap and port together to activate the device.** |  |  |  |  |  |  |
| 1. **Pinches the “skin” at the injection site to form a tent.** |  |  |  |  |  |  |
| 1. Holds the port of the device while inserting the needle. |  |  |  |  |  |  |
| 1. Inserts the needle into the tent of skin between the thumb and forefinger. |  |  |  |  |  |  |
| 1. Inserts the needle at a downward angle. |  |  |  |  |  |  |
| 1. Inserts the needle completely so that the port is in full contact with the skin. |  |  |  |  |  |  |
| 1. Moves fingers from the port to the reservoir “bubble” while still pinching the skin. |  |  |  |  |  |  |
| 1. **Squeezes the reservoir slowly to inject — taking about 5–7 seconds.** |  |  |  |  |  |  |
| 1. Removes the device from the injection site while still pinching skin. |  |  |  |  |  |  |
| 1. Does not rub the injection site. |  |  |  |  |  |  |
| 1. Places the used device immediately into a sharps disposal container without replacing the needle cap. |  |  |  |  |  |  |
| **Total the number of ‘Ss’ (steps completed correctly) for each attempt and enter the total in the columns at right.**  **Circle any of the critical steps (bold) that the woman does NOT do correctly.** |  |  |  |  |  |  |

| In your clinical judgment, has this individual demonstrated sufficient competence to self-inject independently AND did the woman do all the 5 critical steps (bold) correctly? YES NO  **If NO, do NOT give a Sayana Press unit to take home, but DO conduct the post-injection interview. Her participation in the study ends following the interview. DO provide a thank you gift.** |
| --- |
